# Supplementary material for: Change of Amino Acid Residues in Idiotypic Nanobodies Enhanced the Sensitivity of Competitive Enzyme Immunoassay for Mycotoxin Ochratoxin A in Cereals
Source: Toxins (Basel). 2020 Apr 23;12(4):273. doi: 10.3390/toxins12040273 (PMC7232238; doi:10.3390/toxins12040273)
Supplement: Supplementary file 1 [file toxins-12-00273-s001.pdf]

Supplementary Materials: Change of Amino Acid Residues in Idiotypic Nanobodies Enhanced the Sensitivity of Competitive Enzyme Immunoassay for Mycotoxin Ochratoxin A in Cereals

Caixia Zhang, Weiqi Zhang, Xiaoqian Tang, Qi Zhang, Wen Zhang and Peiwu Li.

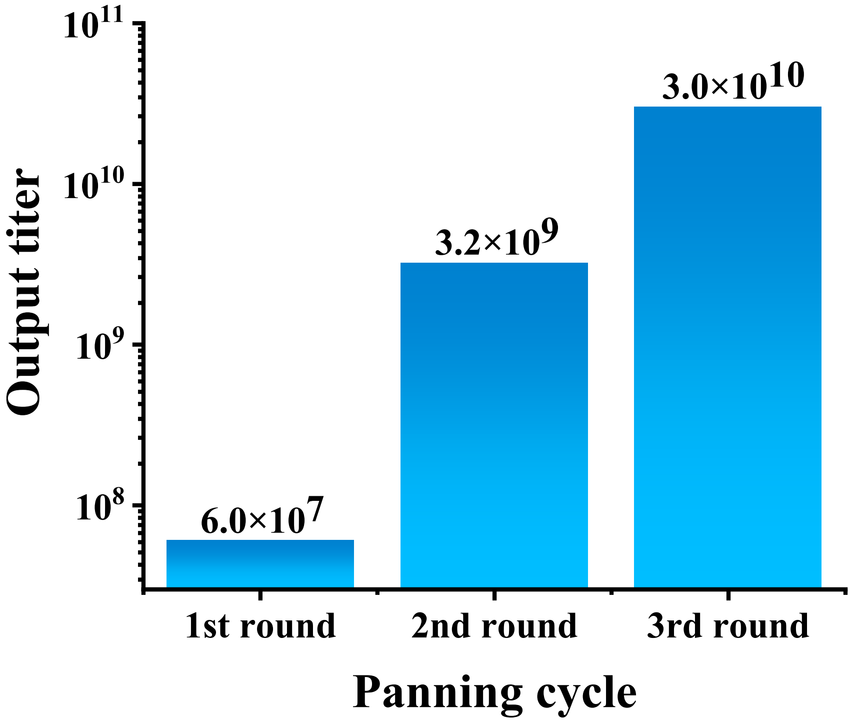

Figure S1. The titer of phage output in three panning cycles. The titer of phage was enriched with the procedure of panning, which indicat the portion of phage with positive effect against mAb 1H2 was enriched.

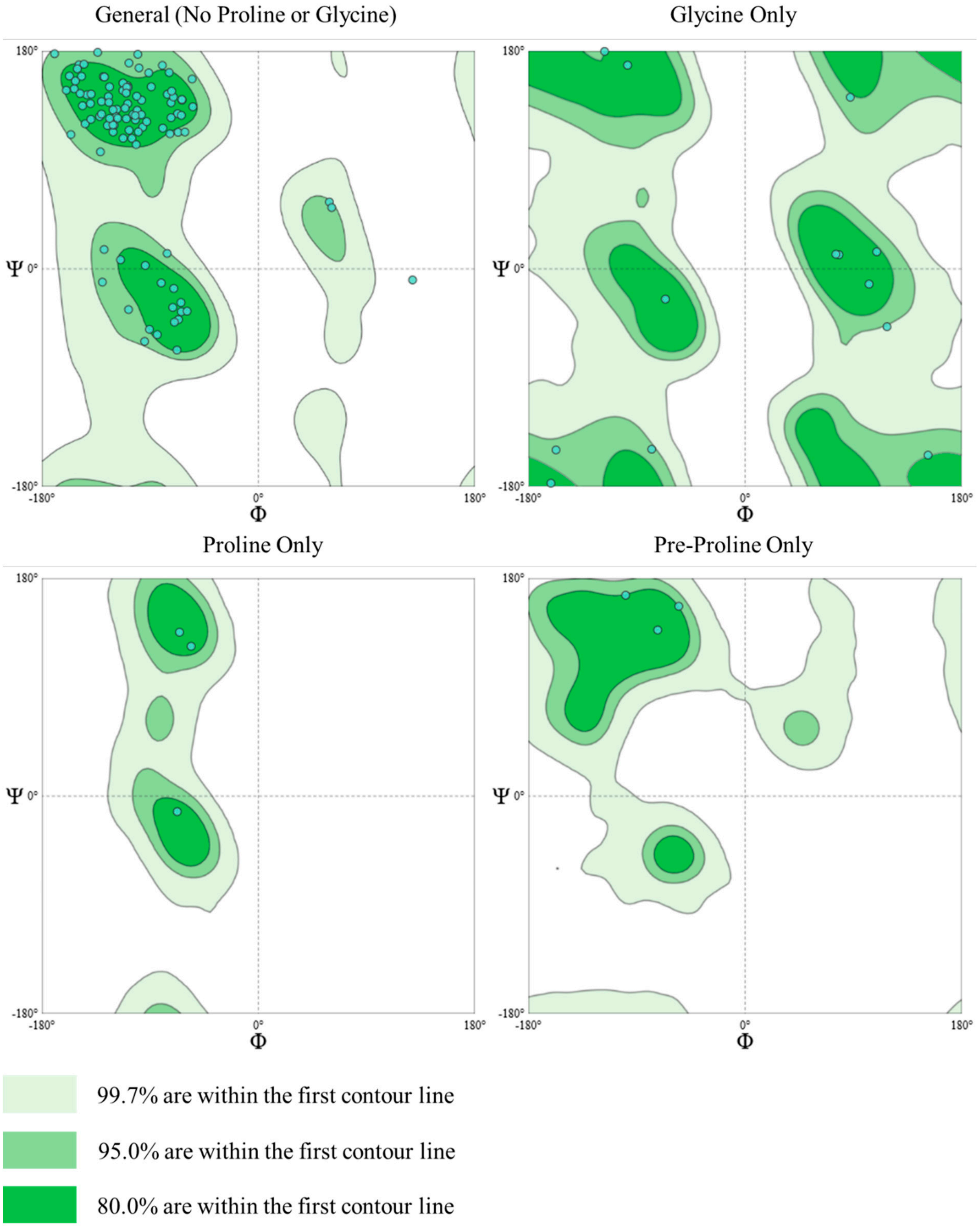

Figure S2. The Ramachandran plots of nontoxic coating antigen 1 (NCA1). The most of backbone dihedral angles against of amino acid residues in NCA1 structure were located in allowed region.

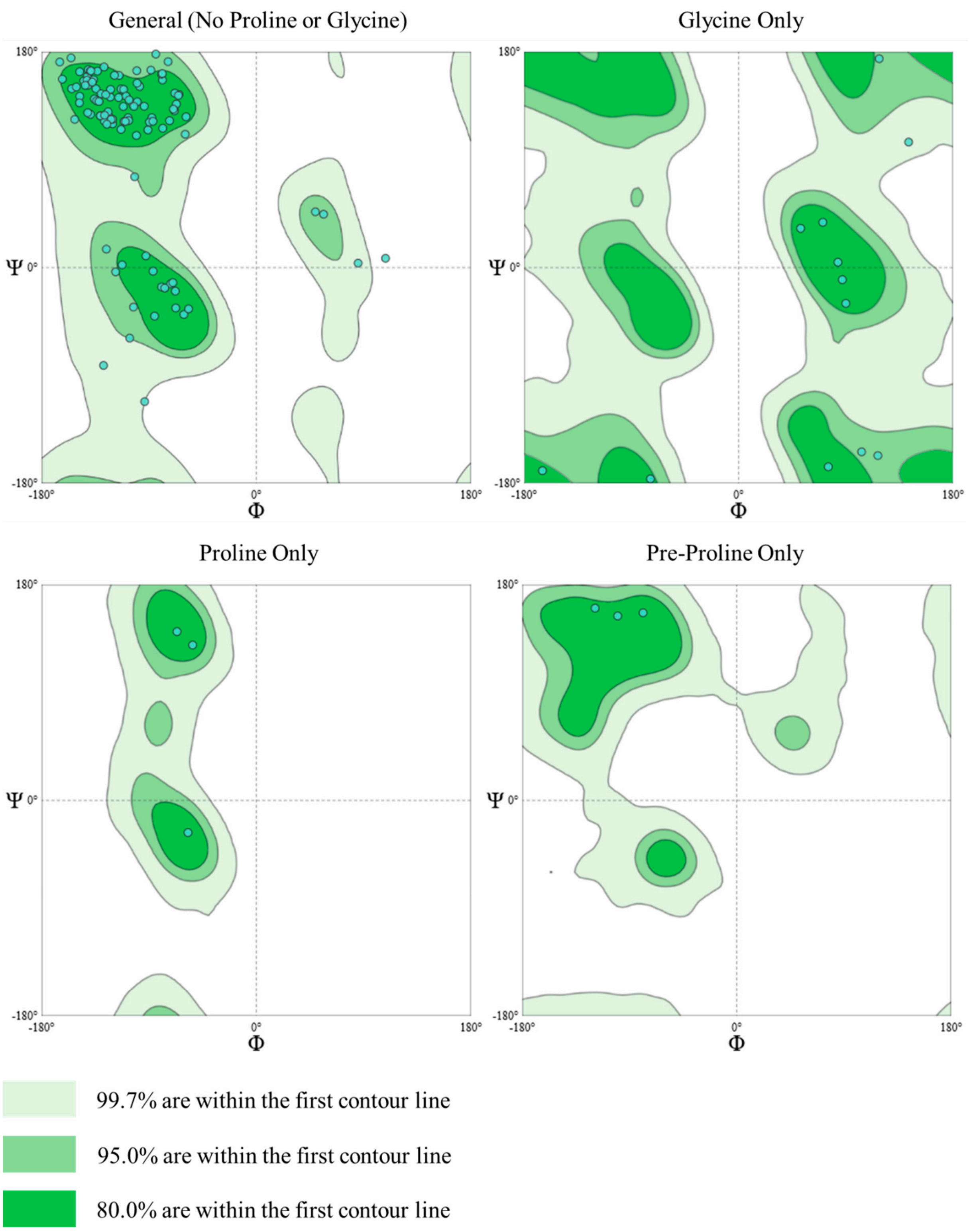

Figure S3. The Ramachandran plots of nontoxic coating antigen 2 (NCA2). The most of backbone dihedral angles against of amino acid residues in NCA2 structure were located in allowed region.

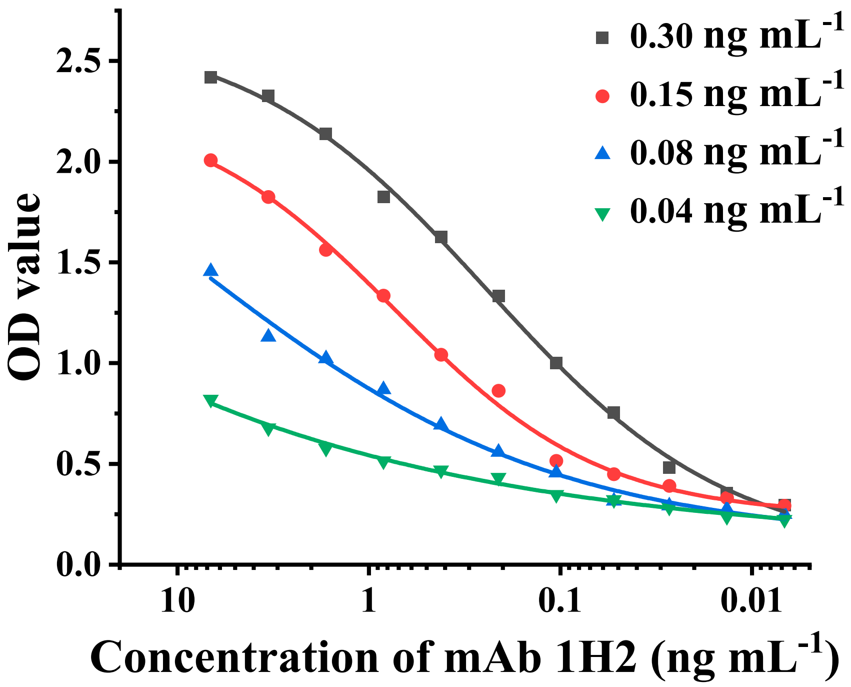

Figure S4. The affinity curve of NCA1. The affinity curve of NCA1 was measured under the NCA1 concentration of 0.3, 0.15, 0.08, and 0.04 ng mL^{-1}. The affinity of NCA1 were the mean value of affinity constants obtained in three kind of concentration ratio of NCA1.

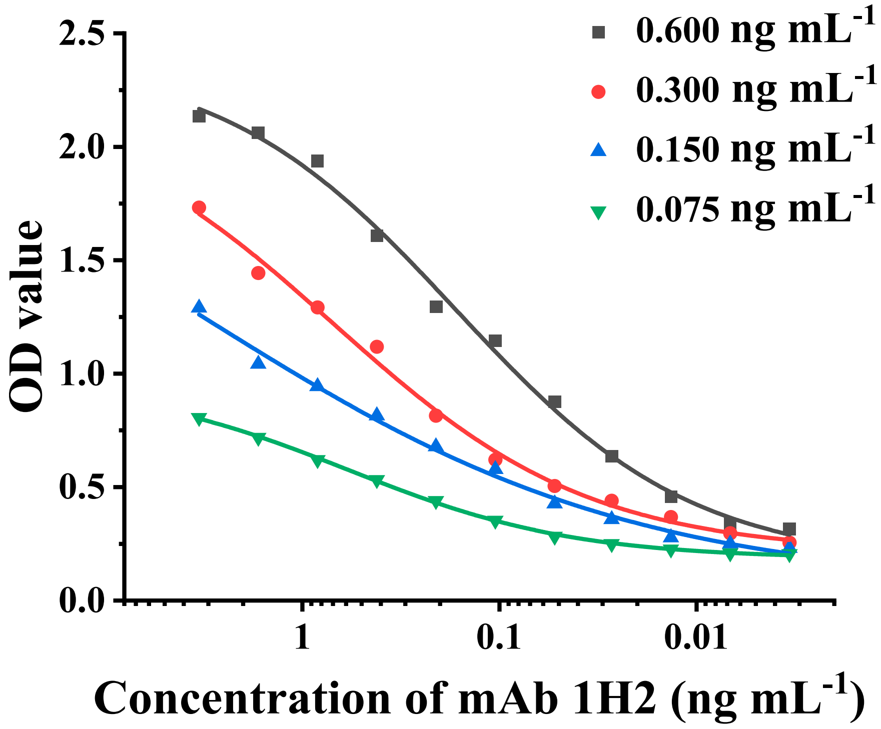

Figure S5. The affinity curve of NCA2. The affinity curve of NCA2 was measured under the NCA2 concentration of 0.6, 0.3, 0.15, and 0.075 ng mL⁻¹. The affinity of NCA2 were the mean value of affinity constants obtained in three kind of concentration ratio of NCA2.

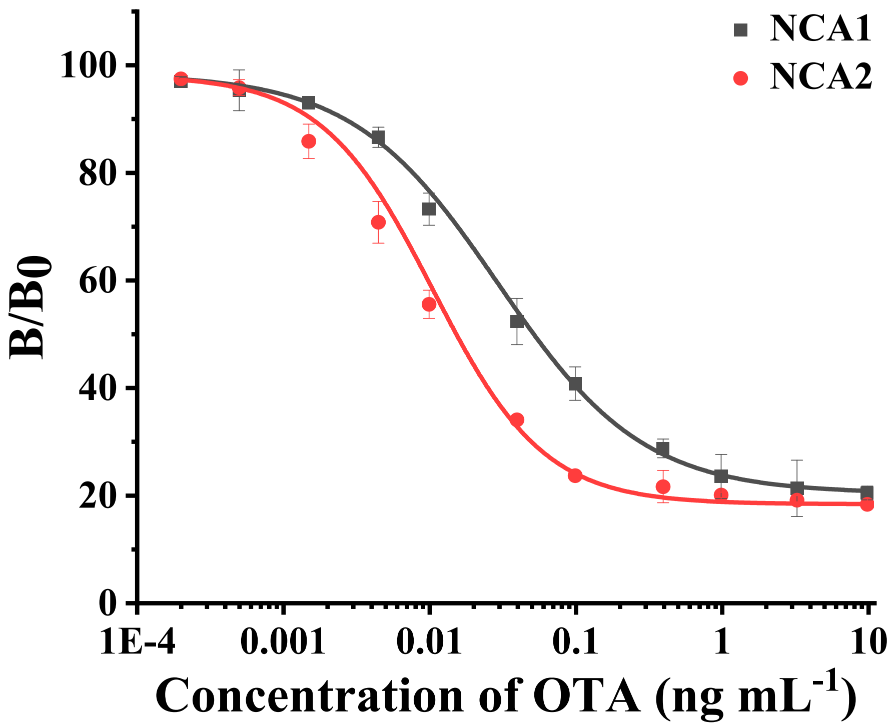

Figure S6. Competitive ELISA with NCAs under optimal condition. The IC_{50} values of NCA1 and NCA2 was 0.052 and 0.015 ng mL^{-1}, respectively.
